# Ionospheric Weather at Two Starlink Launches during Two-Phase Geomagnetic Storms

**DOI:** 10.3390/s23157005

**Published:** 2023-08-07

**Authors:** Tamara Gulyaeva, Manuel Hernández-Pajares, Iwona Stanislawska

**Affiliations:** 1 The Pushkov Institute of Terrestrial Magnetism, Ionosphere and Radiowave Propagation of the Russian Academy of Sciences (IZMIRAN), Troitsk, Moscow 108840, Russia; 2 Department of Mathematics, Universitat Politècnica de Catalunya—IOnospheric Determination and Navigation Based on Satellite and Terrestrial Systems (UPC-IonSAT), 08034 Barcelona, Spain; manuel.hernandez@upc.edu; 3 Space Research Center, Polish Academy of Sciences, 00-716 Warsaw, Poland; stanis@cbk.waw.pl

**Keywords:** ionosphere, global electron content, GIM, forecast, Starlink satellite launch, geomagnetic storm

## Abstract

The launch of a series of Starlink internet satellites on 3 February 2022 (S-36), and 7 July 2022 (S-49), coincided with the development of two-phase geomagnetic storms. The first launch S-36 took place in the middle of the moderate two-phase space weather storm, which induced significant technological consequences. After liftoff on 3 February at 18:13 UT, all Starlink satellites reached an initial altitude of 350 km in perigee and had to reach an altitude of ~550 km after the maneuver. However, 38 of 49 launched spacecrafts did not reach the planned altitude, left orbit due to increased drag and reentered the atmosphere on 8 February. A geomagnetic storm on 3–4 February 2022 has increased the density of the neutral atmosphere up to 50%, increasing drag of the satellites and dooming most of them. The second launch of S-49 at 13:11 UT on 7 July 2022 was successful at the peak of the two-phase geomagnetic storm. The global ionospheric maps of the total electron content (GIM-TEC) have been used to produce the ionospheric weather GIM-W index maps and Global Electron Content (*GEC*). We observed a *GEC* increment from 10 to 24% for the storm peak after the Starlink launch at both storms, accompanying the neutral density increase identified earlier. GIM-TEC maps are available with a lag (delay) of 1–2 days (real-time GIMs have a lag less than 15 min), so the GIMs forecast is required by the time of the launch. Comparisons of different GIMs forecast techniques are provided including the Center for Orbit Determination in Europe (CODE), Beijing (BADG and CASG) and IZMIRAN (JPRG) 1- and 2-day forecasts, and the Universitat Politecnica de Catalunya (UPC-ionSAT) forecast for 6, 12, 18, 24 and 48 h in advance. We present the results of the analysis of evolution of the ionospheric parameters during both events. The poor correspondence between observed and predicted GIM-TEC and *GEC* confirms an urgent need for the industry–science awareness of now-casting/forecasting/accessibility of GIM-TECs during the space weather events.

## 1. Introduction

Knowledge of the state of the global ionosphere presents an indispensable tool in the planning and operation of space experiments. An example of such space activity is the Starlink satellite network developed by the private spaceflight company SpaceX to provide low-cost internet access to remote locations. In view of increasing solar activity during recent and forthcoming years approaching the peak of the solar cycle 25, the satellites started sinking toward the atmosphere at an unusually fast rate—up to 10 times faster than before [1]. Satellites orbiting close to Earth always face the drag of the residual atmosphere, which gradually slows the spacecraft and eventually makes them fall back to the planet.

Such an event occurred with the Starlink launch on 3 February 2022 (S-36), which happened in the middle of the moderate two-phase space weather storm [2]. After liftoff on 3 February at 18:13 UT, all Starlink satellites reached an initial altitude of 350 km in perigee and had to reach an altitude of ~550 km after the maneuver. However, 38 of 49 launched spacecrafts did not reach the planned altitude, left orbit due to increased drag and reentered the atmosphere on 8 February. A geomagnetic storm on 3–4 February 2022 has increased the density of the neutral atmosphere up to 50%, increasing drag on the satellites and dooming most of them [2,3,4,5,6,7,8]. Tsurutani et al. [6] mentioned the E × B convection lift of the ionospheric electrons and ions to higher altitudes causing the drag. At the same time, photoionization by solar UV and EUV radiation replaces the depleted lower-altitude ions, giving an overall increase in *TEC* on the dayside. To compare the ionospheric effects for the S-36 launch with another similar event, we considered the second launch of S-49 at 13:11 UT on 7 July 2022, which was successful at the peak of the two-phase geomagnetic storm.

The global ionosphere maps of total electron content (GIM-TEC) are used in the present study for the analysis of the ionospheric weather during two Starlink launches. GIM-TEC maps are available with a lag (delay) of 1–2 days, and the real-time GIMs have a lag of 15 min [9]. Hence, the GIMs forecast is required in any case by the time of any satellite launch, because the ‘true’ GIMs are produced only for the time prior to the launch. The goal of the present study was the evaluation of the performance of the different forecast GIMs during the two Starlink launches. The Jet Propulsion Laboratory (JPL)-produced GIM-TEC maps [10] are routinely used to calculate the ionospheric weather GIM-W index maps [11]. W index represents the logarithmic deviation of instantaneous *TEC* from the preceding 15-day sliding median TECμ at the same hour UT for each cell of GIM. The W index has four levels for the positive logarithmic deviation from the quiet state (W = 1) to an intense positive storm (W = 4) in steps of 1 and four levels for the negative deviation from the quiet state (W = −1) to the intense negative storm (W = −4). Thresholds for the different grades of the W index and values of the logarithmic deviations for the positive and negative W indices are given in Table 3 of [11].

Forecasts of GIM-TEC and GIM-W maps for 1 and 2 days in advance (denoted further as d1 and d2) are produced for the current day and the next day by IZMIRAN, from the JPLR GIM-TEC provided daily for the preceding day [11]. Independent 1- and 2-day GIMs forecasts provided by the Center for Orbit Determination in Europe (CODE, CODE1 and CODE2) are also used for the analysis [12]. The third class of 1- and 2-day GIMs forecast (B1PG and B2PG) are provided by the Beijing University of Aeronautics and Astronautics model [13]. The 1- and 2-day GIMs forecasts with the data set CASG provided by the Chinese Academy of Sciences are the fourth data set under consideration [14]. The Universitat Politècnica de Catalunya (UPC-ionSAT) GIMs forecasts for 6, 12, 18, 24 and 48 h in advance present another family of the Nearest-Neighbor (NN) model [15] used for the comparisons. There are other methods of GIMs forecast published in literature which are not used in the present analysis [16,17,18,19,20,21,22,23,24].

As a measure of the instant ionosphere state, we use the Global Electron Content (*GEC*) [25,26]. The global electron content (*GEC*) is equal to the total number of electrons in the near-Earth space environment within the GPS orbital altitude of approximately 20,200 km. It presents unique metrics of the instant ionosphere state providing one magnitude, *GEC* (in GECU units, 1GECU = 10^32^ electrons) derived from the instant Global Ionospheric Map of Total Electron Content (GIM-TEC) [9,10,27,28,29]. While evaluations of the different GNSS global ionospheric mapping techniques for the observed GIMs have been made by different authors [27,28,29], the global view forecast of GIMs is evaluated for the first time in the present study. The results of comparisons of the different GIMs forecast techniques are provided in the following sections.

## 2. Data Processing Results

**Figure 1:**
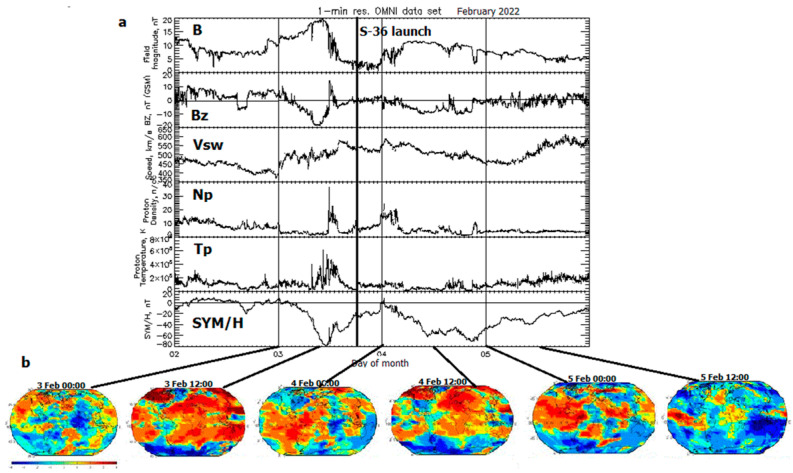
The space weather conditions from 2 to 5 February 2022 under which the Starlink S-36 was launched at 18:13 UT on 3 February (thick vertical line). From top to bottom: (**a**) magnetic field intensity *B*, nT; the southward component Bz, nT; the solar wind speed Vsw, km/s; the proton density Np, cm^−3^; the proton temperature Tp, K; the geomagnetic SYM/H index, nT; (**b**) GIM-W index maps based on UQRG GIM-TEC for 00:00 and 12:00 UT during the storm.

**Figure 2:**
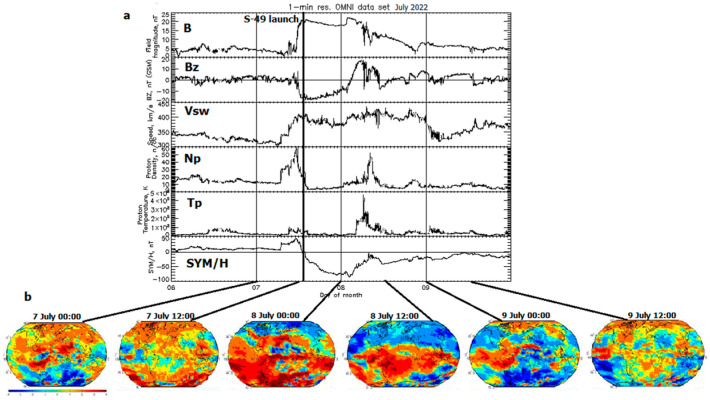
The same as Figure 1a,b but from 6 to 9 July 2022 related with the Starlink S-49 launch at 13:11 UT on 7 July.

The space weather conditions for two two-phase geomagnetic storms occurring at the Starlink launches are illustrated in Figure 1a,b and Figure 2a,b, and the peak values of all parameters are listed in Table 1.

The interplanetary magnetic field (IMF) 5 min parameters provided by OMNI are plotted in Figure 1a from 2 to 5 February 2022. Here, we observe from top to bottom: the IMF magnetic field magnitude *B*, in nT; the field’s southward component Bz, in nT, in the GSM coordinate system; the solar wind speed Vsw, in km/s; the proton density Np, in cm^−3^; the plasma temperature Tp, in K; and the equatorial geomagnetic SYM/H index, in nT. The Starlink S-36 launch (thick vertical line) at 18:13 UT on 3 February 2022 occurred between the 1st peak of the geomagnetic storm (symmetric ring current index SYM-H = −80 nT at 10:55 UT on 3 February) and the 2nd peak (SYM-H = −70 nT at 20:40 UT on 4 February). Note that only one peak is observed in the Tp temperature profile at 11:15 UT on 3 February 2022. Selected GIM-W index maps derived from 15 min tomographic-kriging, UPC rapid GIM (UQRG) GIM-TECs are plotted in Figure 1b for 00:00 and 12:00 UT on 3, 4 and 5 February. The global spatial distribution of W index includes quiet conditions at the prestorm hour 00:00 UT on 3 February. An enhanced positive *TEC* disturbance (red area) is most essential, for example, during the peaks of the geomagnetic storm. The negative storm effects (blue area) are observed over the Antarctic region at the time approaching the storm peaks and became dominant after the 2nd storm peak. Note that the GIM-TEC and GIM-W maps could not be examined in real time during the Starlink operation: they are produced with 1 or 2 days’ lag (delay) after the space weather event when the GIMs became available, so these GIMs can be used only for the postprocessing analysis.

Figure 2a presents IMF parameters similar to Figure 1a provided by OMNI from 6 to 9 July 2022. The Starlink S-49 launch at 13:11 UT on 7 July (thick vertical line) occurred two hours later than the storm onset which is close to the 1st peak of the proton density (Np = 56.84 cm^−3^ at 11:25 UT on 7 July). The geomagnetic storm shows gradual development during 15 h of the main phase ending with the 1st peak SYM-H = −85 nT at 02:15 UT on 8 July, and the 2nd less intense peak was observed with SYM-H = −42 nT at 11:35 UT on 8 July. The gradual development of the geomagnetic storm is accompanied by a quiet GIM-W index (Figure 2b, at 00:00 and 12:00 UT on 7 July). The positive GIM-W enhancement is dominant around the peak of SYM-H (00:00 UT on 8 July), then it is confined at the southern hemisphere towards the storm recovery of SYM-H at 12:00 UT on 8 July. The increased negative W index effects in the northern hemisphere are gradually replaced by the negative storm in the southern hemisphere. Again, these GIMs can be used only for the postprocessing analysis, because they are produced with a lag of 1–2 days from the relevant GIM-TECs.

While we observe the local or regional features of the near-Earth plasma on the global GIMs, an advantage of the global electron content (*GEC*) is the presentation of the state and variability of the ionosphere as a whole. At a given time, the global electron content depends on 3-D electron density distribution, in terms of latitude, longitude and height, integrated over the volume of the ionosphere and plasmasphere from the surface of the Earth to the altitude of GPS satellites [25]. Calculation of the *GEC* proxy is based on GIM-TEC maps available in latitude *φ* from 87.5° S to 87.5° N in steps of Δ*φ* = 2.5°, longitude *λ* from 180° W to 180° E in steps of Δ*λ* = 5°. Individual grid values *TEC* (*φ_i_*, *λ_j_*) from GIM are used to produce *GEC* with Equation (1):

(1) $$GEC=\sum_{i}S_{i}(\varphi_{i})\sum_{j}TEC(\varphi_{i},\lambda_{j})$$

The surface area coefficient *S_i_*(*φ_i_*) for the cells centered at grid [*φ_i_*, *λ_j_*] depends on latitude *φ_i_* and step in longitude Δ*λ*:

(2) $$S_{i}(\varphi_{i})={\left(R_{E}+h\right)}^{2}\cdot \;\left|\Delta \lambda \right|\cdot \;|\sin\left(\varphi_{1}\right)-sin(\varphi_{0})|$$

with

$$\varphi_{1}=\varphi_{i}+\frac{\Delta \varphi }{2},\;\;\varphi_{0}=\varphi_{i}-\frac{\Delta \varphi }{2}$$

The coefficients are determined according to the geocentric distances of the *TEC* receiver and the ionospheric pierce point (in our case, the Earth’s radius *R_E_* = 6370 km plus height *h* = 450 km). The total number of coefficients *S_i_*(*φ_i_*) is equal to 71 (*φ_i_* = −87.5, −85, …, 87.5° N), which are calculated a priori and applied with Equation (1) to different GIMs.

**Figure 3:**
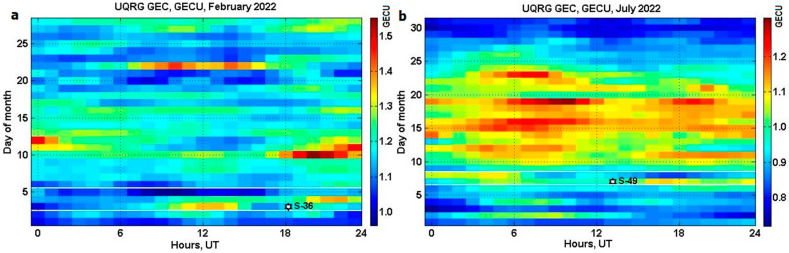
Global electron content (*GEC*) in daily–hourly UT frame produced from the UPC UQRG: (**a**) February 2022; (**b**) July 2022. Starlink S-36 and S-49 launches (star).

Hour-by-hour *GEC* calculations with the above equations from the hourly UPC tomographic-kriging GIM ‘UQRG’ (0, 1, …, 23 UT) for February 2022 (Figure 3a) and July 2022 (Figure 3b) reveal two features of *GEC* variation: (i) missing diurnal *GEC* variation because the local time changes are masked by latitudinal—longitudinal map summation (Equations (1) and (2)); (ii) appreciable periods of enhanced/depleted *GEC*. The Starlink launches (marked with a star) are observed during the moderate geomagnetic storms (denoted with white lines). Every pixel in the images of Figure 3a,b is derived from a separate GIM, so that *GEC* presents a metrics reducing the GIM’s data set by the number of pixels in one GIM, i.e., 5112 times based on the information in the paper.

**Figure 4:**
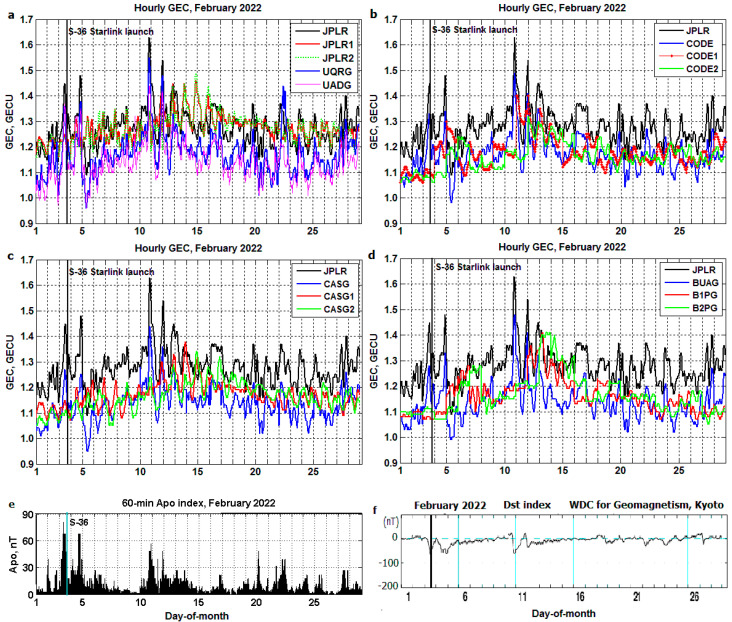
Daily–hourly variation of *GEC* and geomagnetic indices for February 2022. The Starlink S-36 launch (thick vertical line). (**a**) JPLR—based ‘true’ *GEC*, 1- and 2-day forecast (JPLR1/JPLR2) produced by IZMIRAN, ‘true’ UQRG and UPC tomographic-kriging real-time UADG *GEC* products; (**b**) CODE ‘true’ *GEC* and CODE1/CODE2 forecast; (**c**) CASG ‘true’ data and CASG1/CASG2 forecast; (**d**) BUAG ‘true’ and B1PG/B2PG forecast; (**e**) geomagnetic Hpo index; (**f**) equatorial Dst index.

Daily–hourly *GEC* variation produced from the different GIMs during February 2022 are plotted in Figure 4a–d. The variation of geomagnetic Apo index equivalent to the Kp index but on an hourly cadence [30] and Dst index [31] during February 2022 are plotted in Figure 4e,f, respectively. The Starlink S-36 launch is shown with a thick vertical line. The JPLR-based *GEC* is provided in all four panels for comparison with other data. The difference between JPLR ‘true’ *GEC* and 1- and 2-day JPLR-based forecast by IZMIRAN (JPLR1 and JPLR2) [10] brought to light some differences, as they can be seen in Figure 4a. Similar differences are observed between CODE ‘true’ data and CODE1/CODE2 forecast (Figure 4b) [12], CASG ‘true’ data and CASG1/CASG2 forecast (Figure 4c) [14], and BUAG ‘true’ and B1PG/B2PG forecast (Figure 4d) [13]. The UPC-IonSAT produces UQRG and real-time UADG on a 15 min cadence [16,27], but only hourly *GEC* derived from them are used in the present study, similar to other kinds of ‘true’ GIMs. Note the close resemblance of hourly UQRG and real-time UADG-based results (Figure 4a) but the difference of their ‘true’ profiles from JPLR, which is also seen for CODE (Figure 4b), CASG (Figure 4c) and BUAG (Figure 4d) (in agreement with long-term comparisons, Figure 2 of [27], Figure 6 of [28] and Figure 3 of [29]).

It has been noted that the density of the neutral atmosphere was increased up to 50% during the geomagnetic storm on 3–4 February 2022, increasing drag of the satellites and dooming most of them [2,3,4,5,6,7,8]. To check a possible increase of *GEC* magnitude during the storm, we compare the peak magnitude *GEC_max_* after the Starlink launch with the base daily-average quiet value *GEC_av_* on the prestorm day. Table 2 provides these parameters for the different data centers after the Starlink launches on 3 February and 7 July 2022. The increment of the global electron content is calculated in percent, *dGECp*, with Equation (3):

(3) $$dGECp=\frac{{GEC}_{max\;}-{GEC}_{av}}{{GEC}_{av}}\;\times \;100\%$$

We observe in Table 2 that the *GEC* increment varies from 10 to 24%, accompanying the neutral density increase [2,3,4,5,6,7,8] for both events. This characteristic presents a measure of the positive *GEC* intensification for the second phase of the ionosphere storm.

To estimate quantitative agreement/disagreement between the different data, we determine the coefficient of determination (*R*-squared or *R*^2^) as a measure of the difference between the ‘true’ data (*Y*) and forecast of *GEC* (*X*) or between the different ‘true’ data (*X* and *Y*) [32]:

(4) $$R^{2}=1-\frac{\sum_{1}^{m}{\left(X_{i}-Y_{i}\right)}^{2}}{\sum_{1}^{m}{\left(Y_{av}-Y_{i}\right)}^{2}}$$

where

(5) $$Y_{av}=\frac{1}{m}\sum_{1}^{m}Y_{i}$$

The *R*^2^ varies from −∞ to 1 (worst value = −∞; best value = +1), so *R*^2^ = 1 presents the best resemblance of two data sets. This feature makes it easy to allocate a reasonable agreement with *R*^2^ > 0.5.

The root-mean-square (*RMS*) deviation (in GECU) is also calculated:

(6) $$RMS=\sqrt{\frac{1}{m}\sum_{1}^{m}{\left(X_{i}-Y_{i}\right)}^{2}}$$

The results of *R*^2^ and *RMS* calculation for the different monthly sets of *GEC* are provided in Table 3 for February and July 2022. The best agreement between the pairs with *R*^2^ > 0.5 is given in bold. JPLR ‘true’ profiles show an unacceptable difference with all data reviewed except UQRG in July 2022. Variations of ‘true’ profiles are very similar, as can be seen in Figure 4, but the absolute values of JPLR *GEC* are greater than any other ‘true’ profile. Since Yav (Equation (5)) is calculated from JPLR *GEC* (the 1st line in Table 3 for February and the 1st line for July), it yields the negative *R*^2^ (Equation (4)), attesting to the poor agreement with other GIMs. Poor *R*^2^ is also obtained for all other indices regarding JPLR *GEC* in the 1st column of Table 3, when Yav (Equation (5)) is calculated from the other indices.

The ‘true’ data of CODE, BUAG, CASG and UQRG are in agreement during February and July in most cases. The ‘true’ UQRG and UADG show agreement with ‘true’ data of other centers except UQRG~JPLR and UADG~UQRG, but they show no agreement with ‘forecast’ data in February. The agreement is best for UQRG with other data sets in July except for JPLR1 and JPLR2 forecast. The next successful agreement is UADG in July except for JPLR ‘true’, IZMIRAN 1- and 2-day forecasts of JPLR1 and JPLR2, and CODE d1, BUAG d1 and d2, and CASG d1 and d2 forecasts. Though some ‘forecasts’ agree with the ‘true’ UQRG and UADG data, these are not supposed to be used together in practice. In general, there is only one case of the agreement of CASG ‘true’ data with the relevant 1-day (d1) ‘forecast’ in July based on GIMs from the same source. At the same time, we observe an agreement between the different ‘true’~‘true’ data pairs, confirming the previous results of comparisons of the different GIMs [27,28,29].

**Figure 5:**
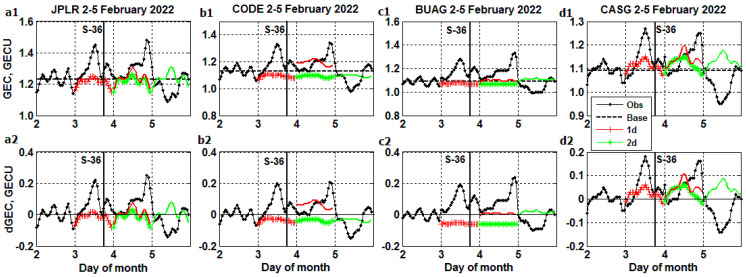
Comparisons of 1- and 2-day forecast with ‘true’ *GEC* profiles during the storm from 2 to 5 February 2022 including the Starlink S-36 launch (thick vertical line): *GEC*—upper panel, detrended *GEC*—lower panel. (**a1**,**a2**) JPLR; (**b1**,**b2**) CODE; (**c1**,**c2**) BUAG; (**d1**,**d2**) CASG.

Recognizing that there are no ‘true’ *GEC* profiles nor ‘true’ GIMs by the time of the Starlink launches, we proceed to the evaluation of GIMs forecast by comparison of *GEC* (as a product of GIM) with ‘true’ *GEC* during two storms. The ‘true’ (Obs.) *GEC* storm profiles are plotted in Figure 5(a1)–(d1) from 2 February (prestorm day) to 5 February 2022: a1—JPLR, b1—CODE, c1—BUAG, d1—CASG. By the time of the Starlink launch at 18:13 UT on 3 February (thick vertical line), the ‘true’ data are available only for the prestorm day. The ‘base’ value presents the average *GEC* during the prestorm day, 1d—1-day forecast for the day of launch, 2d—2-day forecast for the next day after the launch. The 1d (red) and 2d (green) curves with symbols present the forecast starting on the day of the launch, and similar curves without symbols denote the forecast starting on the next day after the launch when the geomagnetic storm is in progress. The detrended *GEC* in Figure 5(a2)–(d2) (after subtraction of the base value from *GEC* ‘true’ and ‘forecast’ data) clearly shows two ‘true’ positive *GEC* storm phases on 3 and 4 February, followed by the negative phase on 5 February. The positive *GEC* enhancements occur at the times of the increased neutral atmosphere density, with increased drag dooming most of the Starlink satellites [2,3,4,5,6,7,8]. We note a failure of *GEC* ‘forecast’ to outline the ‘true’ storm effects observed in Figure 5(a1)–(d2).

**Figure 6:**
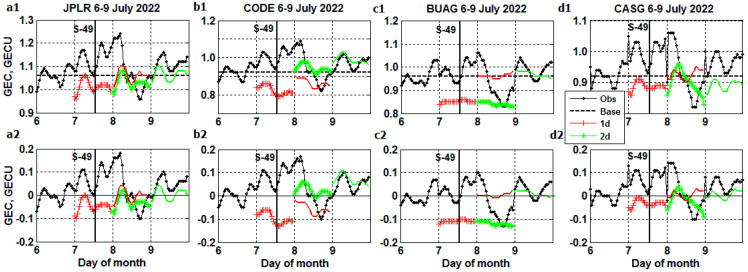
The same as Figure 5 but from 6 to 9 July 2022, including the Starlink S-49 launch.

Similar nonconforming results are obtained between *GEC* ‘true’ and ‘forecast’ profiles plotted from 6 July (prestorm day) to 9 July 2022, including the Starlink S-49 launch at 13:11 UT on 7 July in Figure 6(a1)–(d3). Fortunately, all 53 Starlink S-49 satellites reached this time in their final orbit, because after the failure of S-36 experienced on 3–8 February 2022, the subsequent Starlink launches used a higher initial orbit [2], thereby avoiding a possible drag enhancement. The ‘forecast’ curves neither reproduce the positive phase of the *GEC* storm on 7 July and from 00:00 to 05:00 UT on 8 July nor the negative *GEC* excursion for the rest of the hours on 8 July except for a part of time fit between JPLR~JPLR1 and CODE~CODE1 in the forecast starting one day after the launch, JPLR~JPLR2, CODE~CODE2 and CASG~CASG2 produced on the day of the launch.

The quantitative estimate of a correspondence between the ‘true’ and ‘forecast’ *GEC* storm data with *R*^2^ and *RMS* using Equations (4)–(6) is presented in Table 4 for the storms on 3–5 February and 7–9 July 2022. There is no reasonable conformity between the forecast (*X*) and true (*Y*) data from the four analysis centers all producing *R*^2^ negative results.

**Figure 7:**
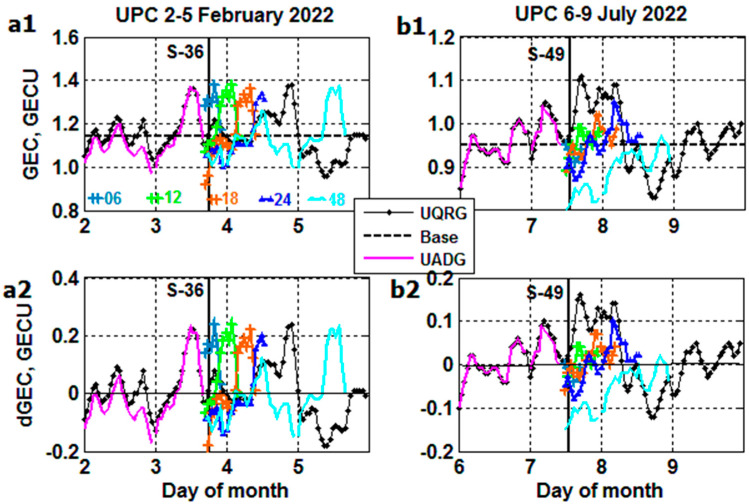
Comparison of ‘true’ UQRG and real-time UADG *GEC* with Nearest-Neighbor (NN) ‘forecast’ for 6, 12, 18, 24 and 48 h in advance. Forecast starts at the time of the Starlink launch (thick vertical line) linked to the real-time UADG data: (**a1**,**a2**) S-36; (**b1**,**b2**) S-49 launch.

The ‘true’ UQRG and real-time UADG results are compared with the Nearest-Neighbor (NN) method of ‘forecast’ for 6, 12, 18, 24 and 48 h in advance [15]. The results are plotted in Figure 7(a1,a2) from 2 February (prestorm day) to 5 February, and in Figure 7(b1,b2) from 6 July (prestorm day) to 9 July 2022. The Starlink launches of S-36 and S-49 are shown by a thick vertical line. An outstanding feature of Figure 7(a1,b2) is the usage of ‘true’ real-time UADG data observed −15 min prior to the launch with ‘forecast’ from 6 to 48 h ahead starting afterwards. We see the diversity of NN forecasts, which differ from the ‘true’ storm profiles failing to fit the positive and negative *GEC* ‘true’ excursions.

**Figure 8:**
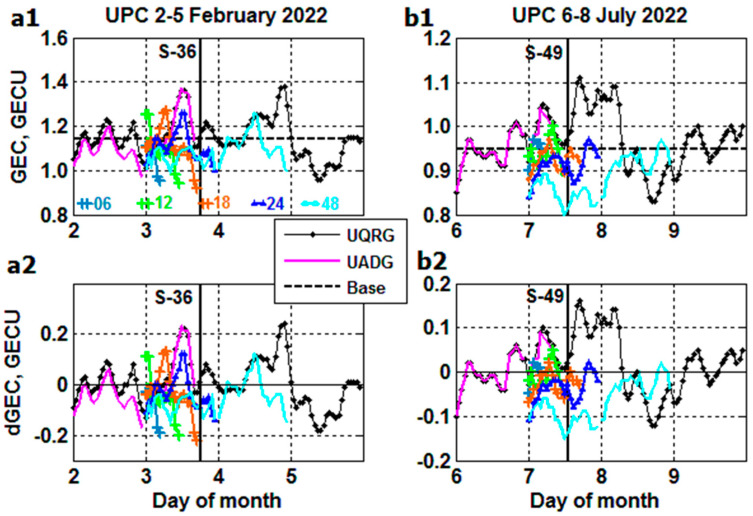
The same as Figure 7 but forecast start at 00:00 UT on the day of the Starlink launch linked to the prestorm day of UQRG.

As distinct from Figure 7(a1)–(b2), the same NN forecasts are plotted in Figure 8(a1)–(b2) but using UQRG ‘true’ data for the day before the launch (prestorm day) and fitting 6, 12, 18, 24 and 48 h ‘forecast’ starting from 00:00 UT on the day of the launch. Here, 24 h forecast appears to better reproduce part of the positive storm effect on 3 February (blue curve) and 48 h forecast (cyan) closer to approaching part of the 2nd positive storm peak on 4 February. The other options differ from the ‘true’ storm profiles for both the S-36 and S-49 events.

The numerical values of *R*^2^ and *RMS* for the NN ‘forecast’ (*X*) are produced using Equations (4)–(6) to estimate their consistency with ‘true’ UQRG and UADG data (*Y*) (Table 5) for the storms on 3–5 February and 7–8 July 2022. Though the better fit of forecast to the ‘true’ *GEC* is observed for the part of storm time in Figure 8, the results of Table 5 for the total storm hours during 3–5 February and 7–8 July reveal an appreciable difference between the NN forecast (*X*) and true (*Y*) data according to *R*^2^ negative results.

## 3. Conclusions

The GIM-TEC global ionospheric maps of the total electron content have been used to produce the ionospheric weather GIM-W index maps and Global Electron Content (*GEC*). GIM-TEC maps are available with a lag (delay) of 1–2 days (real-time GIMs have a lag of less than 15 min), so the GIMs forecast is required by the time of the launch of satellites.

We observed a *GEC* increment from 10 to 24% for the storm peak after the Starlink launch at both storms, accompanying the neutral density increase. This characteristic presents a measure of the positive *GEC* intensification for the second phase of the ionosphere storm.

The different 1- and 2-days ‘forecast’ of GIMs produced by IZMIRAN, CODE, CASG and BUAG centers is compared with ‘true’ *GEC* profiles during two geomagnetic storms on 3–5 February and 7–9 July 2022 when S-36 and S-49 Starlink satellites have been launched at storm time. The NN UPC-ionSAT forecast for 6, 12, 18, 24 and 48 h in advance has been also evaluated for these events.

A numerical estimate of performance of forecasts is made with R-square (*R*^2^) and *RMS* formulae. The monthly estimates of *R*^2^ for February and July 2022 reveal either the acceptable conformity or the difference between the ‘true’ and ‘forecast’ GIMs transformed to Global Electron Content metrics. However, a similar estimate for the storm conditions on 3–5 February and 7–9 July 2022 disclosed a failure to reasonably forecast *GEC* (and GIMs). While the positive *R*^2^ = 1 presents the best conformity of the data we obtained, the negative *R*^2^ for all forecasting results during the storms which characterize unreliable storm-time performance of the forecasting techniques under consideration. The RMS deviations during the storm are greater than those obtained for the total month of February and of July, where the storm conditions are mixed with the dominant quiet times.

The predicted GIMs providing closer forecasted *GEC* (and detrended *GEC*) to the now-casted ones are those with horizons at 24 (and 48) h, and from the day after the storm, i.e., once the now-casted GIMs have experienced the starting of the space weather event. The results of this study aim to revisit the GIM prediction, from the *GEC* perspective, for understanding, firstly, and improving, afterwards, the corresponding forecasting, specially at the subdaily horizons.

Variabilities of the Earth’s ionosphere during the storms can adversely affect the space-based technological infrastructures, such as Low-Earth Orbit (LEO) satellites including the Starlink network and the Global Navigation Satellite System (GNSS). In turn, the GNSS observations and GIMs products provide a ground for the development of reliable GIMs forecasting techniques, which presents a challenge for the satellites’ operation during the space weather events.

## Figures and Tables

**Table 1:** Peak values of space weather indices during two Starlink launches.

Event	Launch 3 February 2022 18:13 UT						Launch 7 July 2022 13:11 UT					
Index	1st Storm Peak			2nd Storm Peak			1st Storm Peak			2nd Storm Peak		
	DD	UT	Peak	DD	UT	Peak	DD	UT	Peak	DD	UT	Peak
B, nT	03	10:15	19.79	04	08:00	11.63	07	13:55	20.97	08	01:45	22.21
Bz, nT	03	09:35	−18.56	04	09:30	−10.0	07	13:50	−17.4	08	11:00	−8.34
Vsw, km/s	03	14:05	579.0	04	00:55	588.1	07	13:50	407.8	08	07:20	423.1
Np, cm^−3^	03	11:45	32.41	04	00:30	24.33	07	11:25	56.84	08	08:10	47.59
Tp, K	03	11:15	4.7 × 10^5^	−	−	−	−	−	−	08	06:35	4.2 × 10^5^
SYM-H, nT	03	10:55	−80	04	20:40	−70	08	02:15	−85	08	11:35	−42

**Table 2:** Peak *GEC* after Starlink launch and its increase in dGEC (%), regarding the daily mean ‘base’ *GEC_av_* for the prestorm day.

Data Center		JPLR	CODE	BUAG	CASG	UQRG	UADG
	**Date**	**3 February 2022**					
*GEC_av_*	2 February 2022	1.23	1.13	1.09	1.09	1.14	1.09
*GEC_max_*	4 February 2022	1.48	1.34	1.33	1.25	1.38	1.35
*dGECp*, %		20.3	18.6	22.0	14.7	21.0	23.9
	**Date**	**7 July 2022**					
*GEC_av_*	6 July 2022	1.06	0.92	0.96	0.92	0.95	0.92
*GEC_max_*	8 July 2022	1.24	1.09	1.06	1.06	1.11	1.08
*dGECp*, %		17.0	18.5	10.4	15.2	16.8	17.4

**Table 3:** *R*^2^ and *RMS* deviations for the different pairs of GIMs during February and July 2022.

	JPLR			Code			BUAG			CASG			UQRG	UADG	
*X*	Obs	d1	d2	Obs	d1	d2	Obs	d1	d2	Obs	d1	d2	Obs	Obs	
***Y***	**February 2022**														
JPLR	−	−0.05 0.073	−0.22 0.079	−1.35 0.109	−2.20 0.126	−2.82 0.138	−3.01 0.142	−3.04 0.142	−3.08 0.143	−3.13 0.144	−2.22 0.127	−2.69 0.136	−1.40 0.110	−3.41 0.149	*R* ^2^ *RMS*
CODE	−1.57 0.109	−2.31 0.123	−2.42 0.126	−	−0.25 0.076	−0.20 0.074	**0.667** **0.039**	−0.374 0.080	−0.68 0.088	**0.631** **0.041**	0.074 0.065	−0.12 0.072	**0.684** **0.038**	**0.681** **0.041**	*R* ^2^ *RMS*
BUAG	−3.24 0.142	−3.84 0.152	−3.96 0.154	**0.679** **0.039**	−0.52 0.085	−0.42 0.082	−	−0.58 0.087	−0.98 0.097	**0.839** **0.028**	−0.20 0.076	−0.27 0.078	0.359 0.055	**0.705** **0.038**	*R* ^2^ *RMS*
CASG	−4.05 0.144	−4.57 0.152	−4.64 0.152	**0.589** **0.041**	−0.73 0.084	−0.43 0.077	**0.813** **0.028**	−0.70 0.084	−1.07 0.092	−	−0.27 0.072	−0.29 0.073	0.248 0.056	**0.674** **0.037**	*R* ^2^ *RMS*
UQRG	−0.89 0.110	−1.59 0.128	−1.68 0.131	**0.772** **0.038**	−0.21 0.088	−0.19 0.087	**0.521** **0.055**	−0.42 0.095	−0.56 0.100	**0.514** **0.056**	−0.02 0.081	−0.12 0.085	−	**0.565** **0.053**	*R* ^2^ *RMS*
UADG	−2.67 0.140	−3.92 0.152	−3.38 0.153	**0.604** **0.041**	−0.40 0.087	−0.25 0.082	**0.739** **0.038**	−0.38 0.086	−0.71 0.096	**0.750** **0.037**	−0.13 0.078	−0.25 0.082	0.484 0.053	−	*R* ^2^ *RMS*
	**July 2022**														
JPLR	−	−5.58 0.916	−55.5 0.953	−0.14 0.135	−4.27 0.291	−1.23 0.189	−0.31 0.145	−0.76 0.168	−0.98 0.178	−0.48 0.154	−0.52 0.156	−0.82 0.171	**0.844** **0.141**	−0.66 0.163	*R* ^2^ *RMS*
CODE	−0.18 0.135	−5.53 0.814	−44.9 0.843	−	−0.67 0.161	−0.56 0.082	**0.561** **0.082**	**0.931** **0.033**	0.479 0.090	**0.945** **0.029**	**0.647** **0.074**	0.301 0.104	**0.992** **0.029**	**0.917** **0.036**	*R* ^2^ *RMS*
BUAG	−0.44 0.145	−5.53 0.809	−46.9 0.835	**0.927** **0.033**	−0.67 0.156	**0.533** **0.082**	−	0.438 0.090	0.241 0.105	**0.907** **0.037**	**0.589** **0.077**	0.186 0.109	**0.984** **0.040**	**0.886** **0.041**	*R* ^2^ *RMS*
CASG	−0.68 0.154	−5.53 0.797	−47.0 0.824	**0.940** **0.029**	−0.51 0.146	**0.579** **0.077**	**0.904** **0.037**	0.370 0.095	0.118 0.112	−	**0.542** **0.081**	0.156 0.109	**0.991** **0.029**	**0.939** **0.029**	*R* ^2^ *RMS*
UQRG	**0.783** **0.146**	−5.51 0.798	−5.35 0.788	**0.982** **0.042**	**0762** **0.153**	**0.925** **0.086**	**0.974** **0.050**	**0.898** **0.100**	**0.859** **0.118**	**0.983** **0.041**	**0.925** **0.086**	**0.884** **0.107**	−	**0.982** **0.042**	*R* ^2^ *RMS*
UADG	−0.85 0.163	−5.47 0.788	−45.2 0.815	**0.910** **0.036**	−0.32 0.138	**0.574** **0.078**	**0.885** **0.041**	0.334 0.098	0.059 0.116	**0.940** **0.029**	0.483 0.086	0.084 0.115	**0.991** **0.029**	−	*R* ^2^ *RMS*

**Table 4:** *R*^2^ and *RMS* deviations for the different pairs of *GEC* observed and relevant 1- and 2-day forecast during geomagnetic storms on 3–5 February and 7–9 July 2022 starting from the day of the Starlink launches.

*X*	JPLR1	JPLR2	CODE1	CODE2	BUAG1	BUAG2	CASG1	CASG2	
***Y***	**3–5 February 2023**								
JPLR	−0.41 0.106	−0.61 0.113							*R* ^2^ *RMS*
CODE			−1.50 0.133	−1.31 0.128					*R* ^2^ *RMS*
BUAG					−1.25 0.125	−0.45 0.100			*R* ^2^ *RMS*
CASG							−0.62 0.101	−0.46 0.095	*R* ^2^ *RMS*
	**7–9 July 2022**								
JPLR	−1.25 0.097	−1.68 0.106							*R* ^2^ *RMS*
CODE			−4.98 0.153	−0.99 0.088					*R* ^2^ *RMS*
BUAG					−1.54 0.096	−2.47 0.112			*R* ^2^ *RMS*
CASG							−1.00 0.086	−2.67 0.116	*R* ^2^ *RMS*

**Table 5:** *R*^2^ and *RMS* deviations between UQRG and UADG data and NN forecast for 6, 12, 18, 24 and 48 h in advance during geomagnetic storms on 3–5 February and 7–8 July 2022 starting from the day of the Starlink launches.

*X*	6 h	12 h	18 h	24 h	48 h	
***Y***	**3–5 February 2022**					
UQRG	−1.18 0.149	−2.53 0.190	−1.68 0.166	−2.71 0.195	−1.52 0.160	*R* ^2^ *RMS*
UADG	−0.39 0.201	−0.75 0.225	−0.67 0.220	−1.107 0.248	−43.8 1.142	*R* ^2^ *RMS*
	**7–8 July 2022**					
UQRG	−0.17 0.086	−0.32 0.092	−0.71 0.104	−3.60 0.170	−2.40 0.147	*R* ^2^ *RMS*
UADG	−0.19 0.081	−0.29 0.084	−0.72 0.097	−3.95 0.164	−2.38 0.136	*R* ^2^ *RMS*

## Data Availability

Jet Propulsion Laboratory (JPL) data base: https://sideshow.jpl.nasa.gov/pub/iono_daily/IONEX_rapid/ (accessed on 31 July 2023). IZMIRAN provides W-index maps and forecasts JPLR1 and JPLR2: https://www.izmiran.ru/ionosphere/weather/ (accessed on 31 July 2023). The Universitat Politècnica de Catalunya (UPC-ionSAT) GIMs: http://cabrera.upc.es/upc_ionex_GPSonly-RINEXv3/ (accessed on 31 July 2023). Center for Orbit Determination in Europe (CODE) data are used from: https://cddis.nasa.gov/archive/gnss/products/ionex/ (accessed on 31 July 2023). GIMs forecast (B1PG and B2PG) are provided by the Beijing University of Aeronautics and Astronautics: http://pub.ionosphere.cn/prediction/daily/ (accessed on 31 July 2023). GIMs forecast with the CASG data provided by the Chinese Academy of Sciences: ftp://ftp.gipp.org.cn/product/ionex/ (accessed on 31 July 2023). OMNI data: https://omniweb.gsfc.nasa.gov/form/omni_min.html (accessed on 31 July 2023). Dst and SYM/H indices: http://wdc.kugi.kyoto-u.ac.jp/ (accessed on 31 July 2023). Apo index: https://kp.gfz-potsdam.de/en/hp30-hp60 (accessed on 31 July 2023).
